# Validation of the Italian Tinnitus Questionnaire Short Form (TQ 12-I) as a Brief Test for the Assessment of Tinnitus-Related Distress: Results of a Cross-Sectional Multicenter-Study

**DOI:** 10.3389/fpsyg.2018.00065

**Published:** 2018-01-31

**Authors:** Roland Moschen, Alessandra Fioretti, Alberto Eibenstein, Eleonora Natalini, Domenico Cuda, Giuseppe Chiarella, Gerhard Rumpold, David Riedl

**Affiliations:** ^1^University Clinic of Medical Psychology, Medical University of Innsbruck, Innsbruck, Austria; ^2^Tinnitus Center, European Hospital, Rome, Italy; ^3^Department of Applied Clinical and Biotechnological Sciences, University of Aquila, L’Aquila, Italy; ^4^Department of Otorhinolaryngology, Guglielmo da Saliceto Hospital, Piacenza, Italy; ^5^Department of Experimental and Clinical Medicine, Unit of Audiology and Phoniatrics, Magna Græcia University, Catanzaro, Italy

**Keywords:** Tinnitus Questionnaire, TQ 12, validation, tinnitus, tinnitus distress

## Abstract

**Objectives:** The use of reliable and valid psychometric tools to assess subjectively experienced distress due to tinnitus is broadly recommended. The purpose of the study was the validation of the Italian version of Tinnitus Questionnaire 12 item short form (TQ 12-I) as a brief test for the assessment of patient reported tinnitus-related distress.

**Design:** Cross-sectional multicenter questionnaire study.

**Setting:** Tinnitus Center, European Hospital (Rome), the Department of Otorhinolaryngology, “Guglielmo da Saliceto” Hospital (Piacenza), and the Department of Audiology and Phoniatry, “Mater Domini” University Hospital (Catanzaro).

**Participants:** One hundred and forty-three outpatients with tinnitus treated at one of the participating medical centers.

**Main Outcome Measures:** Tinnitus Questionnaire Short Form (TQ 12-I), compared to the Tinnitus Handicap Inventory (THI), Brief Symptom Inventory (BSI), and Short Form (SF-36) Health Survey.

**Results:** Our factor analysis revealed a two-factor solution (health anxiety, cognitive distress), accounting for 53.5% of the variance. Good internal consistency for the total score (α = 0.86) and both factors (α = 0.79–0.87) was found. Moderate correlations with the THI (*r* = 0.65, *p* < 0.001) indicated good convergent validity. Tinnitus distress was further correlated to increased psychological distress (*r* = 0.31, *p* < 0.001) and reduced emotional well-being (*r* = -0.34, *p* < 0.001).

**Conclusion:** The study clearly showed that the TQ 12-I is a reliable and valid instrument to assess tinnitus-related distress which can be used in clinical practice as well as for research.

## Introduction

Tinnitus is defined as a subjective acoustic perception in the absence of any external source (Hallam et al., 1984). Epidemiological studies reported a prevalence of 10–16% for chronic tinnitus in the adult population that increases with age (Davis and El Refaie, 2000; Shargorodsky et al., 2010; McCormack et al., 2014). The majority of people with tinnitus do not suffer from it strongly. Yet, about 0.5–3% of the general population develop severe distress and experience impairment in everyday life, sleep, mood, concentration and daily work (Davis and El Refaie, 2000; Gopinath et al., 2010). Because the subjectively experienced distress due to tinnitus cannot sufficiently be explained by psychoacoustic parameters (e.g., tinnitus loudness) (Henry and Meikle, 2000), psychological factors like depression, anxiety or catastrophizing have been assumed to be adjuvant to explain tinnitus distress (Milerová et al., 2013; Weise et al., 2013; McKenna et al., 2014).

National and international guidelines recommend using psychometrically and clinically validated questionnaires for the assessment of tinnitus severity (Langguth et al., 2007; AWMF, 2015). In order to be applicable during routine clinical practice a questionnaire for the assessment of tinnitus severity should not only be psychometrically and clinically validated but also quickly administered to minimize the patients’ burden. Several reliable and internationally validated tools for the assessment of tinnitus severity have been developed. One of the most commonly used tools is the Tinnitus Questionnaire (TQ) (Goebel and Hiller, 1998), which assesses different aspects of tinnitus related distress (emotional and cognitive distress, intrusiveness, auditory perceptual difficulties, sleep disturbance and somatic complaints). The TQ has been translated and validated in several different languages, including German, Dutch, French, and Chinese. To enable a faster but equally reliable assessment of tinnitus related distress the TQ 12 was initially developed by Hiller and Goebel (2004). The TQ 12 was validated in German (Hiller and Goebel, 2004), Portuguese (Cerejeira et al., 2009), Greek (Panagiotopoulos et al., 2015), Chinese (Kam, 2012), Dutch (Vanneste et al., 2011), and Arabic (El Beaino and Eter, 2017). Good psychometric properties were reported for all of the translated versions of the TQ 12: the psychometric analyses across various validations showed good reliability (internal consistency: α = 0.86–0.90; retest-reliability: *k* = 0.89–0.91) and high correlation with the TQ total score (*r* = 0.90–0.93) (Hiller and Goebel, 2004; Kam, 2012; Zeman et al., 2012; Panagiotopoulos et al., 2015). The TQ 12 total score allows the classification of the patients as compensated (0–7), moderately distressed (8–12), severely distressed (13–18), and most severely distressed (19–24) (Hiller and Goebel, 2004). Yet, conflicting evidence was found for the factorial structure: most of the validations presumed a single global factor, whereas Panagiotopoulos et al. (2015) reported a three factor solution (Distress, Health pre-occupation, and Depression). So far, neither the TQ nor the TQ 12 have been validated in Italian. The aim of the present study was to evaluate the reliability, validity and factorial structure of the Italian TQ 12-I.

## Materials and Methods

### Sample and Setting

The sample of this cross-sectional multi-center study consisted of 143 outpatients, which were include from the following healthcare institutions: Tinnitus Center, European Hospital (Rome), the Department of Otorhinolaryngology, “Guglielmo da Saliceto” Hospital (Piacenza), and the Department of Experimental and Clinical Medicine, Unit of Audiology and Phoniatrics, University “Magna Graecia” (Catanzaro). Patients were included in the study if they (a) had tinnitus for at least 3 months, (b) were older than 18 years, (c) spoke Italian fluently and (d) had no apparent cognitive impairment. The patients completed the questionnaires as part of the routine clinical practice. Written informed consent was obtained by all patients.

### Measures

#### Tinnitus Sample Case History (TSCH)

Sociodemographic and clinical data were assessed with the Italian version of the TSCH. It was developed by the Tinnitus Research Initiative as in attempt to standardize the assessment of sociodemographic and clinical data in tinnitus research (Langguth et al., 2007). The questionnaire consists of 35 items on background (i.e., age, gender), tinnitus history (i.e., loudness, pitch, percentage of awake time aware of tinnitus, hyperacusis) and related conditions (i.e., hearing impairment, noise annoyance, vertigo/dizziness).

#### Tinnitus Questionnaire (TQ 12)

The TQ 12 consists of 12 items, which can be scored on a three point ordinal Likert Scale ranging from 0 to 2. The total score has a range of 0–24 points with higher values indicating higher tinnitus distress. The items of the TQ 12 correlated highly with the TQ total score, were reliable and showed good responsiveness (Hiller and Goebel, 2004). The Italian translation of the English TQ 12 was provided by an Italian native speaker, fluent in the source language and expert in the medical field of tinnitus. This forward translation was checked by a second independent person with the same level of expertise.

#### Tinnitus Handicap Inventory (THI)

The Tinnitus Handicap Inventory (Newman et al., 1996) is a one of the most commonly used questionnaires to assess tinnitus distress. The THI consists of 25 items that can be divided in a functional, emotional and catastrophic scale. Patients can score the frequency/intensity of these symptoms on a three point ordinal Likert Scale ranging from 0 to 4. The total score ranges from 0 to 100 points with higher values indicating higher tinnitus distress The total score the THI can be graduated in five grades of tinnitus severity: slight (0–16), mild (18–36), moderate (38–56), severe handicap (58–76), and catastrophic (78–100) (Zeman et al., 2014). Good reliability (α = 0.94) and validity were reported for the total score of the Italian THI version (Passi et al., 2008; Salviati et al., 2013).

#### Brief Symptom Inventory (BSI)

Mental health symptoms and psychological distress were assessed with the Brief Symptom Inventory (BSI; Derogatis, 1993). The BSI consists of 53 items, which can be divided into nine subscales and three scales to capture global psychological distress. Good reliability and validity for the subscales and total score were reported (De Leo et al., 1993).

#### Short Form (36) Health Survey

The health status was measured with the Short Form (36) Health Survey (SF-36) (Ware and Sherbourne, 1992), a broadly used, well established instrument to assess the Health-Related Quality of Life (HRQOL). The SF-36 consists of 36 items which can be divided into eight subscales and a physical and psychological total score. The subscale’s internal consistencies were tested in nine samples, showing a Cronbach’s α between 0.77 and 0.93. Good validity has been reported in several studies (Apolone and Mosconi, 1998).

### Statistical Analysis

Psychometric values (means, standard deviations, item-total correlations) of the items are presented. The floor and ceiling effect, defined as the highest and lowest 15% of the scale were also calculated. A factor analyses (maximum likelihood, direct oblimin) with a fixed number of factors was conducted to investigate the three-factor solution proposed by Panagiotopoulos et al. (2015). Based on the results of this initial analysis an additional exploratory factor analysis was calculated. Scree plots and Eigenvalues were used to determine the ideal number of factors. Reliability was evaluated by calculating Cronbach’s α (internal consistency) and item-total correlations. The convergent validity was evaluated by two-sided Pearson correlations with the THI and the discriminant validity was examined by correlations with BSI and SF-36 scores. To test if the proposed TQ 12-I severity grades differentiated well enough between different levels of tinnitus distress, analyses of variances (ANOVAs) with Bonferroni-corrected *post hoc* analyses were calculated. Statistical analyses were conducted using IBM SPSS (v.22).

## Results

A total sample of 146 patients were included in the study, of which 46.5% were recruited in Piacenza, 36.1% in Rome, and 17.4% in Catanzaro. The sample had a mean age of 53.6 years and 54% were men. The mean tinnitus duration was 7.5 years and a large proportion of the sample reported tinnitus in both ears (41.0%). More than half of the sample (57.5%) had subjective hearing problems and about a third (36.3%) reported physical discomfort because of surrounding sounds (hyperacusis). About 21% of the sample had undergone several or many treatments due to their tinnitus. Based on the THI total score patients’ tinnitus distress was graded as follows: 19.9% very mild, 26.2% mild, 31.9% moderate, 15.6% severe, and 6.4% catastrophic. About 5% of the sample reported to be currently under treatment for psychiatric disorders. For details see **Table 1**.

**Table 1:** Clinical properties of the sample.

TN loudness: 0–100 (*SD*)	53.2 (28.9)
Missing	10 (6.8%)
Duration: years (*SD*)	7.5 (10.3)
Family history of tinnitus complaints	37 (25.3%)
Missing	1 (0.7%)
Subjective hearing problem	84 (57.5%)
Missing	1 (0.7%)
Hearing aid	19 (13.7%)
Missing	1 (0.7%)
Hyperacusis	53 (36.3%)
Missing	1 (0.7%)
Tinnitus loudness varies during the day:	
Yes	96 (65.8%)
No	48 (32.9%)
Missing	2 (1.4%)
Pulsatile tinnitus	41 (27.5%)
If yes: with heartbeat	15 (38.5%)
If yes: different from heartbeat	24 (61.5%)
Location	
Right ear	30 (20.5%)
Left ear	36 (24.7%)
Both ears	60 (41.0%)
Inside the head	11 (7.5%)
Missing	9 (6.2%)
Pitch	
Very high frequency	20 (13.7%)
High frequency	58 (39.7%)
Medium frequency	49 (33.6%)
Low frequency	12 (8.2%)
Missing	7 (4.8%)
Number of treatments for tinnitus	
None	78 (53.4%)
One	37 (25.3%)
Several	23 (15.8%)
Many	6 (4.1%)
Missing	2 (1.4%)

### Psychometric Values

The mean, standard deviation and item-total correlation of each item are presented in **Table 2**. The mean TQ 12-I total score was 10.8 (*SD*: 5.8). No floor or ceiling effect was found.

**Table 2:** Mean, standard deviations, and variance of the TQ 12-I.

	*Mean*	*SD*	Item-total correlation
1. I am aware of the noises from the moment I get up to the moment I sleep	1.34	0.75	0.14
2. Because of the noises I worry that there is something seriously wrong with my body	0.61	0.80	0.50
3. If the noises continue my life will not be worth living	0.48	0.80	0.44
4. I am more irritable with my family and friends because of the noises	0.82	0.72	0.66
5. I worry that the noises might damage my physical health	0.74	0.76	0.61
6. I find it harder to relax because of the noises	0.99	0.74	0.65
7. My noises are often so bad that I cannot ignore them	1.14	0.75	0.57
8. It takes me longer to get to sleep because of the noises	0.99	0.82	0.53
9. I am more liable to feel low because of the noises	0.90	0.78	0.71
10. I often think about whether the noises will ever go away	1.19	0.80	0.32
11. I am a victim of my noises	0.73	0.82	0.62
12. The noises have affected my concentration	0.92	0.76	0.70

*Item-total correlations were corrected for the individual contribution of each item*.

**FIGURE 1:**
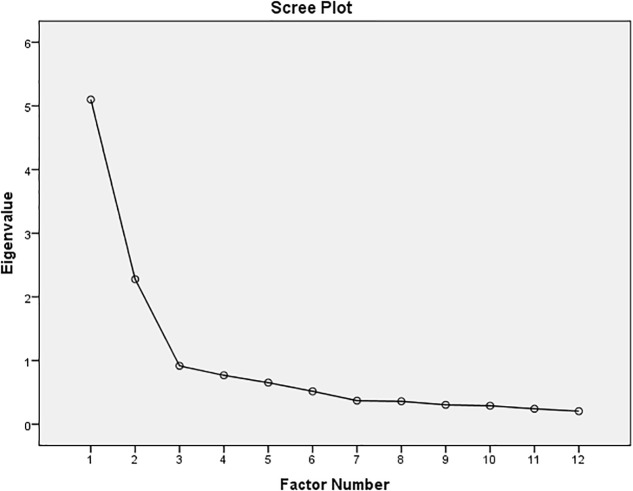
Scree plot for the TQ 12-I.

We initially tested the proposed factor solution of Panagiotopoulos et al. (2015) by factor analysis (main component analysis and maximum likelihood). Bartlett’s test of sphericity [χ^2^ (66) = 827.9, *p* < 0.001] was significant and the Kaiser–Meyer–Olkin measure verified the sampling adequacy for the analysis (KMO = 0.85). Initial Eigenvalues indicated that the first three factors explained 41.7%, 19.1%, and 7.9% of the variance, respectively. The three-factor-solution explained 58.4% of the variance, the diagonals of the anti-image correlation matrix were between 0.69 and 0.93. The factorial structure was examined with oblique rotation (oblimin), but we were not able to replicate the proposed three-factor-solution. Furthermore, the scree plot and the Eigenvalues supported a two-factor-solution (**Figure 1**). Therefore we also calculated an exploratory factor analysis (maximum likelihood) without a predefined number of factors. The results clearly supported the two-factor solution, explaining 53.5% of the variance. **Table 3** shows the factor loadings after rotation. Based on these results the remaining calculations were conducted for the two-factor solution. The content analysis for naming the extracted factors was independently conducted by two researchers, differences were resolved by consensus (reconciliation process). The items clustered on the same component suggest that factor 1 represents ‘health anxiety,’ while factor 2 represents ‘cognitive distress’ related to tinnitus.

**Table 3:** Results of the factor analysis (Pattern Matrix).

	Factor	
	Health anxiety	Cognitive distress
1. I am aware of the noises from the moment I get up to the moment I sleep	-0.188	**0.534**
2. Because of the noises I worry that there is something seriously wrong with my body	**0.765**	0.054
3. If the noises continue my life will not be worth living	**0.827**	-0.025
4. I am more irritable with my family and friends because of the noises	**0.654**	0.490
5. I worry that the noises might damage my physical health	**0.751**	0.232
6. I find it harder to relax because of the noises	0.409	**0.776**
7. My noises are often so bad that I cannot ignore them	0.217	**0.812**
8. It takes me longer to get to sleep because of the noises	0.414	**0.508**
9. I am more liable to feel low because of the noises	**0.639**	0.508
10. I often think about whether the noises will ever go away	0.080	**0.460**
11. I am a victim of my noises	**0**.**700**	0.311
12. The noises have affected my concentration	0.531	**0.715**

*Extraction Method: Maximum Likelihood; Rotation Method: Oblimin with Kaiser Normalization; bold values indicate which factor the item was assigned*.

Good internal consistency was found for the total score (α = 0.86) as well as for factor 1 ‘health anxiety’ (α = 0.87) and factor 2 ‘cognitive distress’ (α = 0.79). The item-total correlations were acceptable for all items, except item 1. Also, the Cronbach’s α would be slightly higher for the total scale if item 1 was excluded (α = 0.87), but not for factor 2.

### Validity Analysis

The TQ 12-I correlated moderately with the THI-total score and its subscales, which indicated good convergent validity. Furthermore elevated tinnitus distress measured by the TQ 12-I total score was correlated positively with higher global psychological distress (BSI total score) and negatively with emotional well-being (SF-36 emotional score). Details are presented in **Table 4**. The correlations with the BSI and SF-36 were significantly lower than with the THI, which indicates good discriminant validity. No significant correlation was found between tinnitus distress and physical well-being.

**Table 4:** Correlations between TQ 12-I, THI, BSI, and SF-36.

	TQ 12-I total score	TQ 12-I health anxiety	TQ 12-I cognitive distress
THI total score	0.65***	0.44***	0.66***
THI functional subscale	0.60***	0.39***	0.64***
THI emotional subscale	0.58***	0.42***	0.56***
THI catastrophic subscale	0.59***	0.41***	0.61***
BSI total score	0.31***	0.21**	0.32***
SF-36 – physical subscale	-0.14	-0.07	-0.17
SF-36 – emotional subscale	-0.34***	-0.22**	-0.36***

*^∗^p < 0.05, ^∗∗^p < 0.01, ^∗∗∗^p < 0.001, two-tailed*

We found no correlation between the TQ 12-I total score and age (*p* = 0.57), gender (*p* = 0.23) or symptom duration (*p* = 0.72). Yet, patients who had higher scores on the TQ 12-I also reported a subjectively louder ear noise (*r* = 0.27, *p* = 0.002). The presence of subjective hearing problems were more present in patients with higher age (*p* = 0.04). In our sample patients with subjective hearing problems did not differ in their perceived tinnitus loudness from patients without subjective hearing problems (*p* = 0.41).

**FIGURE 2:**
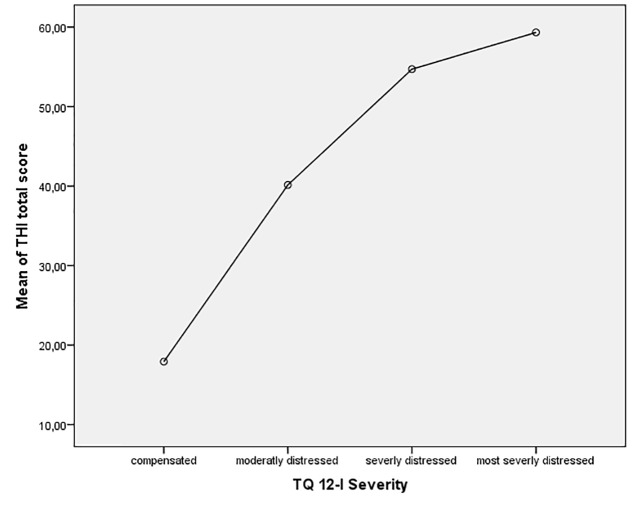
Comparison of TQ 12-I severity groups with THI total score.

According to the proposed cut-off values by Hiller and Goebel (2004) for the German TQ 12 31.9% of the present sample reported no clinically relevant tinnitus distress (1–7 points), 27.0% were moderately distressed (8–12 points), 32.6% were severely distressed (13–18 points) and 8.5% were most severely distressed (>19 points). The ANOVA showed significant differences between the TQ 12-I severity groups regarding the THI-total score (*F* = 31.86, *p* < 0.001). *Post hoc* analyses revealed significant differences between all severity groups, except severely and most severely distressed patients (**Figure 2**).

## Discussion

For use in busy clinical practice, a questionnaire for the assessment of tinnitus severity should not only be psychometrically and clinically validated, but also quickly to use to minimize the patients’ burden. For the TQ 12 good psychometric and clinical values have been reported in several languages (Hiller and Goebel, 2004; Cerejeira et al., 2009; Vanneste et al., 2011; Kam, 2012; Zeman et al., 2012; Panagiotopoulos et al., 2015). Thus far the psychometric properties of the Italian translation of the TQ 12 have not been investigated. The aim of the present study therefore, was to evaluate the Italian short version of the Tinnitus Questionnaire, the TQ 12-I, in a multicenter study in which 143 patients with tinnitus complaints from three study centers participated. The results of our study showed that the TQ 12-I is a reliable and valid measure for assessing tinnitus-related distress and its severity.

The psychometric evaluation of the individual items showed no floor- or ceiling effects, which indicates that all items are well suited to discriminate between low and high levels of tinnitus distress. We found item-total correlations from acceptable to good (values higher than 0.3) for all items, except for item 1 (‘I am aware of the noises from the moment I get up to the moment I sleep’), which had a value of 0.14. This was somehow surprising since item 1 had good item-total correlations (values of 0.3–0.6) in all other versions of the TQ 12 (Hiller and Goebel, 2004; Cerejeira et al., 2009; Vanneste et al., 2011; Kam, 2012; Panagiotopoulos et al., 2015). The translation of the items was independently checked by native-speakers in the course of translation and confirmed by further native speaking researches in the course of the data interpretation. Because of the good psychometric properties in previous studies and the high content validity of the item, we recommend to retain the item in the questionnaire.

It has been pointed out that the factorial structure of the TQ is a weak spot of the otherwise well suited questionnaire. Thus far, only one study has investigated the factorial structure of the TQ 12 (Panagiotopoulos et al., 2015). In that study, with three hundred and one adult Greek patients, the authors reported a three-factor solution, which could not be replicated in our sample. An additional exploratory factor analysis showed that a two-factor solution was better suited for the patients in our sample. Apart from intercultural differences, a possible explanation for the conflicting findings might be the comparatively low mean TQ 12 score in the Greek validation study compared to our study and other validation studies (Hiller and Goebel, 2004; Cerejeira et al., 2009; Kam, 2012).

The two factors were named ‘health anxiety’ and ‘cognitive distress,’ based on a thorough content analysis. The factor **‘health anxiety’** included questions that depicted tinnitus-related anxiety, for example catastrophic assumptions regarding one’s own future life if the tinnitus persisted (item 3 ‘If the noises continue my life will not be worth living’), fears about an underlying severe physical illness (item 2: ‘Because of my noises I worry that there is something seriously wrong with my body’) or about threatening consequences of tinnitus (item 5: ‘I worry that the noises might damage my physical health’). Additionally, the questions also cover the influence of the tinnitus on the patients’ mood (item 9: ‘I am more liable to feel low because of the noises’) and feelings of helplessness or resignation toward tinnitus (item 11: ‘I am a victim of my noises’).

The factor **‘cognitive distress’** includes questions that address experienced impairments in daily life, such as difficulties to distract oneself from the tinnitus (item 7: ‘My noises are often so bad that I cannot ignore them’), to relax or to concentrate (item 6: ‘I find it harder to relax because of the tinnitus’; item 12: ‘The noises have affected my concentration’), or to fall asleep (item 8: ‘It takes me longer to get to sleep because of the noises’).

Several studies showed a close association of distressing tinnitus with anxiety, depressive symptoms and sleep disturbances (Wallhausser-Franke et al., 2012; Cronlein et al., 2016; Ziai et al., 2017). The TQ 12-I captures aspects of tinnitus-related anxiety, catastrophic worrying about its causes and consequences as well as impairments in daily life, mood and functions. Due to its easy and intelligible wording the TQ 12-I can be administered to patients with differing educational backgrounds. The identification and reduction of tinnitus-associated emotional and cognitive distress through patient education, reassurance and demystification of tinnitus promote the habituation process of tinnitus (Moschen et al., 2015). The TQ 12-I provides indications for a subsequent differentiated assessment of comorbid disorders by validated psychopathology questionnaires (Wallhausser-Franke et al., 2012) and may serve for the assessment of therapeutic outcomes.

In our study, the total score of the TQ 12-I had a good internal consistency of α = 0.86, which was comparable with previous studies (Hiller and Goebel, 2004; Cerejeira et al., 2009; Vanneste et al., 2011; Kam, 2012; Zeman et al., 2012; Panagiotopoulos et al., 2015). The internal consistency of the two subscales was also good (health anxiety: α = 0.87; cognitive distress: α = 0.80). The previously problematic item 1 did not lower the factor’s Cronbach α.

We furthermore investigated the validity of the TQ 12-I and its subscales through correlations with tinnitus distress (THI), general psychological distress (BSI), and quality of life (SF-36). As expected, the correlation between the two tinnitus related questionnaires was strong and highly significant, which indicated good convergent validity. Higher tinnitus distress as measured by the TQ 12-I was correlated with higher psychological distress and with lower emotional quality of life. These findings are in accordance with a large body of previous research (Shargorodsky et al., 2010; Milerová et al., 2013; Riedl et al., 2015). The two factors also showed significant correlations with the THI and its subscales as well as psychological distress and emotional quality of life.

In their initial development of the TQ 12 Hiller and Goebel (2004) proposed four grades of tinnitus severity, based on the total score of the TQ 12. To increase the usability of the TQ 12-I in a clinical context we have evaluated the severity grades for the TQ 12-I. Our analysis confirmed the proposed cut-offs, although the differences between the ‘severely distress’ and ‘most severely distressed’ groups were not statistically significant. This might be caused by the comparatively small size of the group with the most severely distressed patients (*n* = 12). The results indicate, that the proposed cut-off values may be used for the Italian TQ 12-I.

### Limitations

In our sample the group of highly distressed patients was comparatively small, although the mean score was comparable to, or even higher than in previous validation studies (Hiller and Goebel, 2004; Vanneste et al., 2011; Panagiotopoulos et al., 2015). The small sample size may have influenced the results of the factor analysis. The proposed factorial structure should be further validated with confirmatory factor analysis in an international sample. In addition, we did not evaluate divergent validity of tinnitus severity with other comorbidity like hearing loss. The cross-sectional design of the present study did not allow us to evaluate the sensitivity to change (responsiveness) or the retest-reliability of the TQ 12-I. Previous studies have shown promising results regarding retest-reliability (Kam, 2012; Panagiotopoulos et al., 2015) and responsiveness (Hiller and Goebel, 2004; Zeman et al., 2012) of the TQ 12.

## Conclusion

The present study showed that the TQ 12-I is a reliable and valid tool to assess the severity of tinnitus distress. The items of the TQ 12-I can be used in everyday clinical practice and research and permit a compact, quick and economical assessment of the most important aspects of subjective tinnitus distress. The provided cutoffs facilitate the interpretation of individual scores and provide indications for a multidisciplinary therapeutic approach.

## Ethics Statement

All procedures performed in studies involving human participants were in accordance with the ethical standards of the institutional and/or national research committee and with the 1964 Helsinki declaration and its later amendments or comparable ethical standards. The study was performed in three ENT centers in Italy, namely Rome, Catanzaro, and Piacenza. Heads of these centers (AE, GC, and DC) agreed on the procedures and provided access to tinnitus patients. Participation was voluntary and every patient gave written consent before filling out the questionnaires.

## Author Contributions

RM and AF designed the study; GC collected the data at the ENT center in Catanzaro; AF, EN, and AE collected the data at the Tinnitus Center in Rome; DC collected the data at the ENT center in Piacenza; AF and EN consulted on the redaction of the manuscript at all stages and contributed to the revision of the manuscript; RM, GR, and DR analyzed the data, wrote the paper and commented on the manuscript at all stages; DR provided the statistical expertise and revised the manuscript at several stages.

## Conflict of Interest Statement

The authors declare that the research was conducted in the absence of any commercial or financial relationships that could be construed as a potential conflict of interest.
